# Assessment of Anger and Burnout Levels Among Addiction Service Operators in Calabria and Sicily: An Open Trial Study

**DOI:** 10.3390/healthcare13131586

**Published:** 2025-07-02

**Authors:** Francesco Principato, Vincenzo Maria Romeo

**Affiliations:** 1 Provincial Health Authority of Reggio Calabria, Via Diana n° 3, 89100 Reggio Calabria, Italy; fraprincipato@gmail.com; 2 Department of Culture and Society, University of Palermo, Viale Delle Scienze, Ed. 15, 90128 Palermo, Italy; 3 School of Psychoanalytic and Groupanalytic Psychotherapy S.P.P.G., Via Fontana n° 1, 89131 Reggio Calabria, Italy

**Keywords:** burnout, anger regulation, addiction service workers, emotional exhaustion, workplace interventions

## Abstract

**Background/Objectives:** Burnout and anger are prevalent among healthcare professionals in high-stress environments, particularly in addiction services. This study explores the relationship between burnout and anger among 124 operators working in public addiction services (SERD) in Calabria and Sicily. The objective is to assess how different anger dimensions contribute to burnout and identify protective factors that could inform targeted interventions. **Methods:** The sample consisted of 58 men and 66 women, with a mean age of 39.2 years (SD = 9.8), ranging from 25 to 59 years old. Burnout was measured using the Maslach Burnout Inventory (MBI), assessing emotional exhaustion, depersonalization, and personal accomplishment. Anger was evaluated through the State-Trait Anger Expression Inventory-2 (STAXI-2), examining trait anger, state anger, anger expression (anger-in, anger-out), and anger control. A cross-sectional design was used, with correlation and regression analyses controlling for gender and years of service. **Results:** High levels of burnout, particularly emotional exhaustion and depersonalization, were found. Emotional exhaustion correlated strongly with trait anger, indicating that individuals with a chronic predisposition to anger are more vulnerable to burnout. Suppression of anger (anger-in) significantly predicted depersonalization, exacerbating emotional disengagement from patients. Conversely, anger control acted as a protective factor, helping maintain a sense of personal accomplishment. **Conclusions:** These findings underscore the importance of emotional regulation in mitigating burnout among addiction service workers. Interventions such as emotional regulation training and anger management programs could help reduce psychological distress and promote resilience. Workplace strategies that support emotional well-being may improve both staff retention and patient care quality. Further research should explore longitudinal trends and intervention effectiveness.

## 1. Introduction

The emotional and psychological well-being of healthcare workers, especially those involved in high-stress sectors such as substance abuse treatment, has garnered significant attention over recent years. In particular, burnout and emotional dysregulation, including anger, have been identified as critical factors that may compromise the effectiveness and well-being of professionals in these environments. Healthcare workers who assist individuals with substance use disorders (SUDs) often face multifaceted challenges, including emotionally taxing patient interactions, exposure to traumatic stories, and systemic resource limitations. These factors contribute to a particularly vulnerable group for psychological distress, where the intersection of burnout and anger becomes a crucial area of investigation [1,2,3]. Recent evidence also shows an alarming increase in emotional exhaustion across the broader healthcare workforce, with nearly 60% of U.S. physicians reporting burnout during the post-pandemic period [4]. 

Burnout is broadly defined as a psychological syndrome that arises in response to prolonged exposure to interpersonal stressors at work. It is conceptualized along three primary dimensions: emotional exhaustion, depersonalization, and a diminished sense of personal accomplishment [5]. Emotional exhaustion refers to feelings of being emotionally overextended and depleted of one’s emotional resources, whereas depersonalization involves a cynical and detached response to various aspects of the job. The third dimension, reduced personal accomplishment, captures the tendency of individuals to evaluate themselves negatively in terms of job performance. These components have been shown to interact, leading to a progressive loss of motivation and engagement with work, which can severely impact both personal well-being and job performance [6]. 

In the context of substance abuse rehabilitation services, workers are particularly susceptible to burnout. Numerous studies indicate that addiction professionals experience high levels of emotional exhaustion due to the challenging nature of their work, including frequent relapses in patients, limited therapeutic successes, and systemic inefficiencies [7]. These stressors are compounded by societal stigma toward addiction, which can further alienate workers and reduce their sense of professional accomplishment [8]. Moreover, the exposure to traumatic events and chronic distress among clients suffering from addiction significantly increases the risk of emotional exhaustion and depersonalization in workers [9]. This burnout may not only affect their mental health but also compromise the quality of care provided, as emotionally exhausted staff may struggle to maintain empathy and effective therapeutic relationships [10].

In parallel with burnout, emotional dysregulation, particularly anger, has emerged as an important factor influencing the mental health of healthcare workers. Anger, a complex emotion that can manifest in response to perceived threats or frustration, plays a significant role in both professional and personal domains [11]. The State-Trait Anger Expression Inventory-2 (STAXI-2), developed by [12], is a widely used instrument for assessing anger. It differentiates between state anger (the emotional reaction to a specific situation), trait anger (a stable tendency to experience anger across a variety of situations), and the various ways in which individuals express or suppress anger.

In high-stress work environments, such as substance abuse services, workers may experience recurrent frustration and powerlessness due to the chronic nature of addiction, frequent patient relapses, and structural constraints like insufficient resources or lack of support [13]. Over time, these stressors can culminate in heightened levels of trait anger, where individuals may develop a chronic predisposition to experience and express anger. Studies have shown that anger can exacerbate the emotional exhaustion component of burnout, as workers who struggle to manage their frustration are more likely to feel overwhelmed by the demands of their job [14].

Moreover, the manner in which anger is expressed or suppressed plays a crucial role in the psychological well-being of healthcare workers. Anger-in (the tendency to suppress anger) has been associated with a range of negative mental health outcomes, including depression and anxiety, while anger-out (the external expression of anger) can lead to interpersonal conflicts and reduced job satisfaction [15]. For SERD operators, who must maintain therapeutic relationships with highly vulnerable patients, poorly managed anger can disrupt the care process and exacerbate their own emotional exhaustion. Additionally, research has demonstrated that anger control (the ability to regulate the intensity and expression of anger) is a protective factor against burnout, as individuals who can effectively manage their emotions are better equipped to cope with the demands of stressful work environments [16].

The relationship between burnout and anger in healthcare settings, particularly in addiction services, is underexplored despite the well-documented emotional challenges faced by these workers. The emotional toll of addiction treatment, combined with the high levels of burnout and anger observed in similar high-stress occupations, suggests that SERD operators may be at heightened risk for psychological distress. Burnout and anger not only affect individual well-being but may also influence the quality of care provided to patients, potentially creating a cycle of negative outcomes for both healthcare providers and their clients.

Several studies have addressed the broader emotional impacts on healthcare workers, identifying common emotional responses, including frustration and anger, as reactions to the stressors inherent in their work [17]. These emotional responses can contribute to the gradual buildup of burnout. Healthcare workers may initially experience frustration due to organizational inefficiencies, role ambiguity, or the lack of perceived progress in patient care [18]. Over time, if these frustrations remain unaddressed, they can develop into chronic anger, further compounding feelings of exhaustion and detachment [19].

Moreover, studies have demonstrated the physiological impact of anger and burnout. Chronic stress, combined with the emotional strain of continuous anger suppression or expression, has been linked to adverse health outcomes, including cardiovascular disease, hypertension, and impaired immune function [20]. This highlights the importance of addressing both burnout and emotional dysregulation, such as anger, in healthcare settings to promote not only psychological but also physical health.

Another important aspect to consider is the role of workplace interventions and organizational culture in mitigating burnout and anger. Research suggests that healthcare organizations that foster a supportive environment, provide access to mental health resources, and promote work–life balance can significantly reduce burnout and improve employee well-being. Implementing resilience training, mindfulness-based interventions, and anger management programs may be particularly effective in helping workers manage the emotional challenges associated with addiction treatment [21].

In summary, the current study aims to bridge the gap in understanding the interplay between burnout and anger among SERD operators in Calabria and Sicily. By using the STAXI-2 and MBI, this research seeks to provide a comprehensive assessment of the emotional and psychological well-being of workers in addiction services, identifying potential areas for intervention and support. Given the high levels of emotional exhaustion and anger reported in similar populations, it is crucial to investigate how these two constructs interact in this specific context and what implications they may have for both individual workers and the broader healthcare system.

## 2. Materials and Methods

### 2.1. Study Design

The current study employs a cross-sectional design to assess the levels of burnout and anger among operators of public addiction services (SERD) in Calabria and Sicily. Cross-sectional studies are frequently used in psychological research to provide a snapshot of the population at a specific point in time, which allows for the identification of correlations and potential risk factors within the sample [22]. Given the objective of this research—to explore the relationship between burnout and anger in a highly specialized healthcare setting—this methodology was considered appropriate for capturing the relevant psychological constructs.

The decision to focus on workers in SERD services was motivated by previous literature, which has demonstrated that healthcare professionals involved in addiction services are exposed to unique stressors compared to other medical and psychological fields. Substance use disorder (SUD) treatment is associated with high levels of emotional labor, the need for constant empathy, and frequent exposure to traumatic or challenging patient narratives, all of which contribute to burnout and emotional dysregulation [3,8]. Furthermore, the regions of Calabria and Sicily were chosen because of their specific socioeconomic and healthcare challenges, which can exacerbate the stress experienced by addiction professionals [13].

### 2.2. Participants

The recruitment process involved contacting addiction service centers (SERD) across Calabria and Sicily through their institutional coordinators. A total of 140 healthcare professionals were invited to participate. Of these, 124 agreed and completed the study in full, resulting in a response rate of 88.57%. A total of 124 operators from various SERD centers across Calabria and Sicily were recruited for the study. The inclusion criteria specified that participants must be employed in a SERD for a minimum of one year and be directly involved in patient care or service management. Participants included nurses, social workers, psychologists, and administrative staff, as these roles are all integral to the multidisciplinary nature of addiction treatment [23]. Exclusion criteria were established to omit participants who had been in their role for less than a year or who were not directly engaged with patients, to ensure that the sample was representative of those most likely to experience burnout and emotional dysregulation due to direct patient interaction [24].

**Figure 1:**
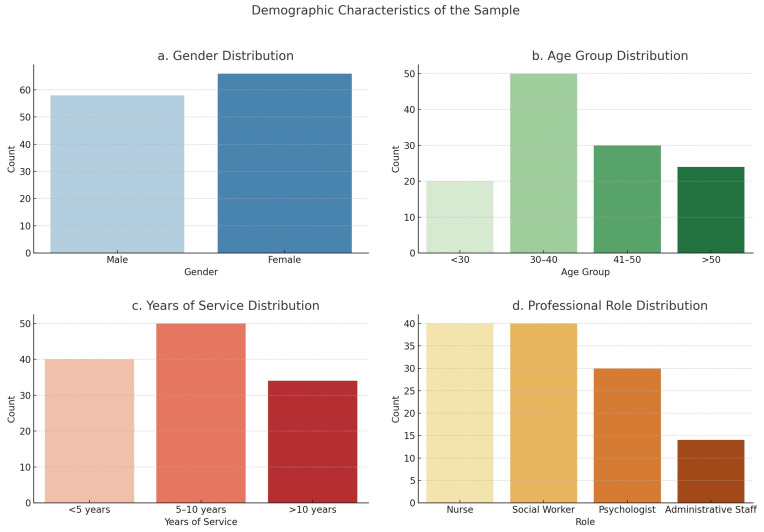
Socio-demographic distribution.

The sample was composed of 58 males and 66 females, with a mean age of 39.2 years (SD = 9.8). The participants’ average length of service in the addiction field was 7.6 years (SD = 3.4), reflecting a broad range of professional experience (Figure 1). Gender, age, professional role, and length of service were recorded to explore whether these variables moderate the relationships between anger and burnout, as demographic factors have been shown to influence burnout levels in previous research [5,25]. For example, studies have demonstrated that females in healthcare are more likely to experience emotional exhaustion than males, which could lead to differences in burnout profiles between genders [26]. 

### 2.3. Instruments

Prior to participation, all individuals were provided with complete information regarding the aim of the study, the procedures involved, the voluntary nature of participation, and their right to withdraw at any time without penalty. Written informed authorization was obtained from each participant. No identifying information was collected, and data were anonymized prior to analysis.

Two primary instruments were used in this study to measure the psychological constructs of interest: the Maslach Burnout Inventory (MBI) and the State-Trait Anger Expression Inventory-2 (STAXI-2).

### 2.4. Maslach Burnout Inventory (MBI)

The MBI is widely regarded as the gold standard for measuring burnout and has been extensively validated across a variety of professional groups, including healthcare workers [27]. The MBI assesses three distinct dimensions of burnout: emotional exhaustion, depersonalization, and personal accomplishment.

1. Emotional exhaustion (EE) refers to feelings of being emotionally drained and overwhelmed by work demands. This dimension is considered the core component of burnout and has been linked to both mental and physical health issues in healthcare workers [28].
2. Depersonalization (DP) reflects a detached or impersonal response to patients or clients, often manifesting as cynicism or negative attitudes towards others. This dimension is particularly relevant in the context of addiction services, where patients may present with challenging behaviors, potentially exacerbating feelings of depersonalization among staff [29].
3. Personal accomplishment (PA) is the sense of competence and successful achievement in one’s work. A low sense of personal accomplishment has been associated with feelings of inadequacy and dissatisfaction in professional roles [30].

In this study, the Italian version of the MBI was used, which has been validated for use in the Italian healthcare context. The MBI consists of 22 items, rated on a 7-point Likert scale from 0 (“never”) to 6 (“every day”). Higher scores on the emotional exhaustion and depersonalization subscales indicate higher levels of burnout, while lower scores on the personal accomplishment subscale are indicative of burnout.

### 2.5. State-Trait Anger Expression Inventory-2 (STAXI-2)

The STAXI-2 is a well-established tool for measuring both the intensity of anger as an emotional state (state anger) and the frequency with which anger is experienced as a personality trait (trait anger) [12]. The inventory also assesses how anger is expressed or controlled, providing a comprehensive evaluation of anger-related processes.

1. State Anger measures the intensity of angry feelings at a particular moment. This subscale is important for understanding how situational factors in the workplace may elicit immediate emotional responses.
2. Trait Anger evaluates how often individuals feel anger over time, making it a useful measure of chronic emotional tendencies.
3. Anger Expression and Control scales assess how individuals express their anger (either outwardly or inwardly) and their ability to control angry feelings. High levels of anger expression (either anger-out or anger-in) have been linked to negative health outcomes, whereas effective anger control is considered a protective factor against psychological distress [16].

The STAXI-2 has been widely used in healthcare settings to assess emotional regulation, including its application in addiction services [31]. For the purposes of this study, the Italian version of the STAXI-2, validated for use in clinical and occupational settings, was employed. The questionnaire consists of 57 items, rated on a 4-point Likert scale from 1 (“almost never”) to 4 (“almost always”), with higher scores indicating greater difficulties with anger regulation.

### 2.6. Procedure

The study followed a structured and ethical approach to data collection, adhering to the guidelines of the Declaration of Helsinki for research involving human subjects [32]. Prior to data collection, ethical approval was obtained from the ethics committees of both the Calabria and Sicily regional healthcare authorities.

### 2.7. Recruitment and Data Collection

Participants were recruited through a combination of email invitations and face-to-face meetings with SERD center directors. The study was introduced as an opportunity to contribute to the understanding of stress and emotional well-being among addiction service professionals. Participation was voluntary, and all participants provided informed consent prior to enrollment.

Data collection took place over a period of three months, with participants completing the MBI and STAXI-2 questionnaires during scheduled breaks or after their work shifts to minimize interference with their professional duties. On average, completion of both instruments took approximately 30–40 min. To ensure the confidentiality of responses, participants completed the questionnaires anonymously, and no identifying information was collected.

### 2.8. Data Analysis

Data were analyzed using SPSS version 26, employing a combination of descriptive statistics, correlation analyses, and multivariate techniques to assess the relationships between the variables of interest.

### 2.9. Descriptive Statistics

First, descriptive statistics (means, standard deviations, frequencies) were computed for all demographic variables, as well as for scores on the MBI and STAXI-2 subscales. Descriptive analysis allowed for an initial understanding of the sample’s burnout and anger profiles and served as the basis for subsequent inferential analyses.

### 2.10. Reliability Analysis

Cronbach’s alpha coefficients were calculated for each subscale of the MBI and STAXI-2 to assess internal consistency reliability. A Cronbach’s alpha value of 0.70 or higher is generally considered acceptable, indicating that the items within each subscale are measuring the same underlying construct [33].

### 2.11. Correlation Analysis

Pearson’s correlation coefficients were used to explore the relationships between the three dimensions of burnout (emotional exhaustion, depersonalization, and personal accomplishment) and the various subscales of anger measured by the STAXI-2 (trait anger, state anger, anger expression, and anger control). Correlation analysis is a commonly employed method for assessing linear relationships between variables in psychological research.

### 2.12. Multivariate Analysis

To assess the predictive relationships between anger and burnout, multiple regression analyses were performed. In the first model, emotional exhaustion was the dependent variable, and trait anger, state anger, anger expression, and anger control were entered as independent variables. Subsequent models were used to explore predictors of depersonalization and personal accomplishment. Demographic variables (e.g., age, gender, length of service) were included as covariates to control for their potential confounding effects.

### 2.13. ANOVA and Group Comparisons

Analysis of variance (ANOVA) was used to examine whether there were significant differences in burnout and anger levels based on demographic variables such as gender, professional role, and length of service. Post hoc analyses with Bonferroni corrections were conducted to explore significant main effects and interactions.

## 3. Results

The results of this study provide an in-depth analysis of the levels of burnout and anger experienced by SERD operators in Calabria and Sicily, as measured by the Maslach Burnout Inventory (MBI) and the State-Trait Anger Expression Inventory-2 (STAXI-2). The data revealed significant findings in terms of both the prevalence of burnout and anger, as well as the interrelationships between these constructs. The results are presented in four primary sections: descriptive statistics, correlation analyses, regression analyses, and group comparisons.

### 3.1. Descriptive Statistics

#### Burnout Levels

**Figure 2:**
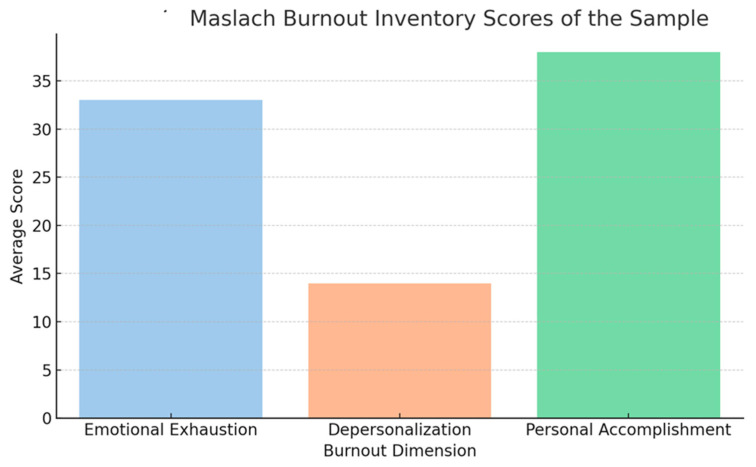
Maslach Burnout Inventory Scores.

Overall, the sample of SERD operators displayed moderate to high levels of burnout, particularly in the domain of emotional exhaustion. The mean score for emotional exhaustion was 32.7 (SD = 8.9), which is consistent with findings from other studies of healthcare professionals in high-stress environments [28,34]. Emotional exhaustion was notably higher in female participants, with an average score of 34.2 (SD = 8.6), compared to 30.9 (SD = 8.9) in males (Figure 2). These findings align with previous research indicating that women in caregiving roles are more prone to emotional exhaustion due to factors such as role conflict, higher expectations for empathy, and societal pressures [26,35]. 

In terms of depersonalization, the mean score was 13.4 (SD = 5.7) (Figure 2), reflecting moderate levels of cynicism and detachment among the participants. Depersonalization was particularly pronounced in operators who had been working in the addiction field for more than 10 years, with a mean score of 15.1 (SD = 5.9) (Figure 2), suggesting that prolonged exposure to patient suffering and relapse may contribute to emotional detachment [29]. Interestingly, no significant differences in depersonalization were observed between genders.

Regarding personal accomplishment, participants reported relatively high levels of perceived efficacy, with a mean score of 37.9 (SD = 6.2). However, those with less than five years of service in addiction treatment reported higher personal accomplishment (M = 39.6, SD = 5.8), compared to those with more than 10 years of service (M = 35.7, SD = 6.4) (Figure 2). This trend suggests that as the length of service increases, workers may experience a decline in their sense of personal accomplishment, which could be attributed to the challenges of treating chronic addiction [7,34].

### 3.2. Anger Levels

The analysis of anger using the STAXI-2 revealed that trait anger was moderately elevated among SERD operators, with a mean score of 23.4 (SD = 6.5), similar to levels reported in other high-stress healthcare settings [16,36]. Participants who scored high on trait anger were found to report frequent feelings of frustration and helplessness in their work, particularly when dealing with patients who exhibited resistance to treatment or frequent relapse episodes.

State anger, or anger experienced in response to specific situations, was also moderately elevated, with a mean score of 19.2 (SD = 4.9). Participants described situations in which they felt anger due to systemic inefficiencies, such as understaffing, lack of resources, and bureaucratic constraints, which align with the broader literature on stress in addiction services [13,37]. 

With regard to anger expression, participants tended to exhibit a preference for anger-in, or the suppression of anger, with a mean score of 18.7 (SD = 5.4). This finding suggests that many SERD operators may internalize their frustration, potentially exacerbating emotional exhaustion and other negative psychological outcomes. Conversely, anger-out, or the outward expression of anger, had a lower mean score of 15.4 (SD = 4.2), indicating that most participants refrained from overt expressions of anger in the workplace. However, those who scored high on anger-out reported experiencing interpersonal conflicts with colleagues, which could further contribute to burnout [18,38]. 

### 3.3. Correlational Analysis

**Figure 3:**
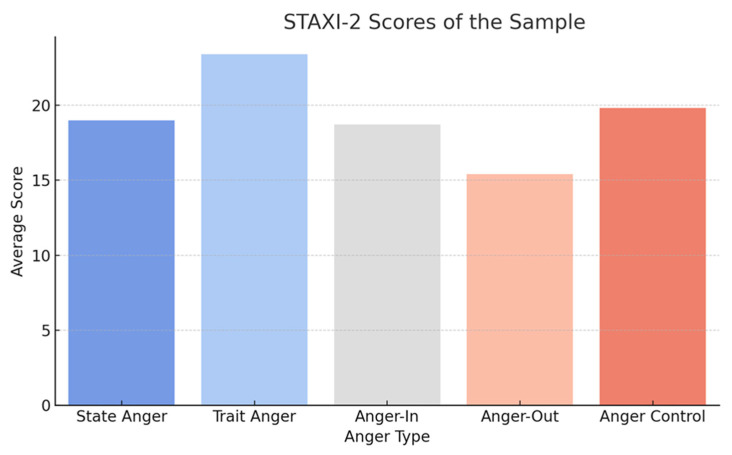
STAXI-2 Scores.

Correlation analyses were performed to explore the relationships between the dimensions of burnout (emotional exhaustion, depersonalization, and personal accomplishment) and the anger variables (trait anger, state anger, anger expression, and anger control). The results demonstrated several significant correlations:

- Emotional exhaustion was positively correlated with trait anger (r = 0.45, *p* < 0.01), indicating that individuals who frequently experience anger across situations are more likely to feel emotionally drained by their work. This supports previous research showing that chronic emotional arousal, such as anger, can exacerbate feelings of fatigue and overwhelm [19,36].
- Depersonalization was significantly correlated with both state anger (r = 0.33, *p* < 0.05) (Figure 3) and anger-in (r = 0.41, *p* < 0.01), suggesting that operators who suppress their anger may become more detached from their patients over time. This finding is consistent with the emotional suppression literature, which posits that suppressing negative emotions can lead to emotional numbness and a reduced capacity for empathy [39].
- Personal accomplishment was negatively correlated with trait anger (r = −0.29, *p* < 0.05) (Figure 3), indicating that individuals with a chronic tendency toward anger are less likely to feel effective in their professional roles. This is in line with studies that have shown how emotional dysregulation undermines self-efficacy and contributes to feelings of inadequacy [40].

**Figure 4:**
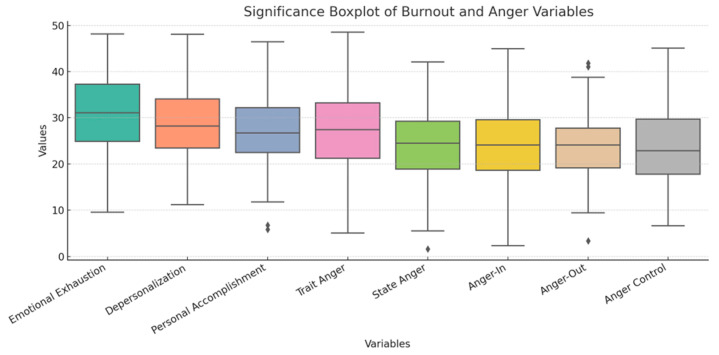
Burnout and Anger correlation. (Rhombuses are outliers—data points that fall outside the whisker range (either below Q1 − 1.5 × IQR or above Q3 + 1.5 × IQR)).

These findings suggest a robust relationship between anger, particularly trait anger and anger-in, and the emotional components of burnout, highlighting the need for interventions that target emotional regulation in this population (Figure 4).

### 3.4. Regression Analysis

Multiple regression analyses were conducted to examine the predictive value of anger variables on burnout dimensions, controlling for demographic variables such as age, gender, and years of service. The results revealed that:

1. Trait anger was a significant predictor of emotional exhaustion (β = 0.36, *p* < 0.01), accounting for 19% of the variance in emotional exhaustion scores. This finding is consistent with prior research that identifies trait anger as a chronic stressor that heightens vulnerability to emotional fatigue [39,41].
2. Anger-in was a significant predictor of depersonalization (β = 0.28, *p* < 0.05), explaining 13% of the variance in depersonalization. This supports the notion that internalizing anger may lead to emotional disengagement and cynicism towards patients, a common feature of depersonalization [29,42].
3. Anger control was a significant negative predictor of personal accomplishment (β = −0.22, *p* < 0.05), suggesting that individuals who are better able to regulate their anger may maintain a stronger sense of efficacy in their work. This finding aligns with studies that have shown the protective role of emotional regulation in maintaining professional competence [16,43]. Figure 1 presents a comparative boxplot displaying the distribution of scores across eight key psychological dimensions assessed in the study: Emotional Exhaustion, Depersonalization, and Personal Accomplishment (derived from the Maslach Burnout Inventory); and Trait Anger, State Anger, Anger-In, Anger-Out, and Anger Control (from the State-Trait Anger Expression Inventory-2).

The boxplots (Figure 3) illustrate the interquartile range (IQR), defined by the 25th and 75th percentiles, with the median score represented by the horizontal line within each box. Whiskers extend to the most extreme values within 1.5 times the IQR, while outliers beyond this range are marked as individual points.

1. Notably, Emotional Exhaustion and Trait Anger display higher median values compared to other variables, indicating a prominent burden of affective dysregulation in the sample. Conversely, Anger Control shows lower central tendency and wider dispersion, suggesting heterogeneity in participants’ ability to regulate anger. The relatively elevated variability in Depersonalization and State Anger further highlights the psychological vulnerability of addiction service professionals exposed to emotionally intense settings.
2. This multidimensional profile underscores the coexistence of burnout symptoms and emotional dysregulation traits, supporting the hypothesis of their interconnectedness and reinforcing the need for targeted emotional regulation strategies in clinical supervision and institutional health programs.

These results (Figure 3) indicate that while anger and burnout are closely linked, different aspects of anger (trait, state, expression) may have differential effects on the various dimensions of burnout. Specifically, trait anger and anger suppression appear to play a central role in exacerbating emotional exhaustion and depersonalization, while anger control is protective against feelings of inefficacy.

### 3.5. Group Differences

ANOVA analyses were conducted to investigate potential group differences in burnout and anger based on gender, professional role, and length of service. The results showed that:

- Gender differences were significant for emotional exhaustion, with females reporting higher levels of exhaustion compared to males (F(1, 122) = 4.67, *p* < 0.05). This finding is consistent with existing literature suggesting that female healthcare workers are more vulnerable to emotional exhaustion due to gendered expectations around caregiving and emotional labor [26,44].
- Professional role also played a significant role in burnout levels. Nurses and social workers reported higher levels of depersonalization compared to psychologists and administrative staff (F(3, 120) = 5.12, *p* < 0.01), which may be attributed to the direct and often intense nature of patient interactions in these roles [25,45].
- Length of service was associated with differences in personal accomplishment, with operators who had been in the field for more than 10 years reporting lower levels of accomplishment compared to those with less experience (F(2, 121) = 6.15, *p* < 0.01). This is in line with previous research suggesting that burnout tends to increase over time in high-stress professions [6,46].

## 4. Discussion

The present study offers an original contribution to understanding the psychological risks faced by operators in addiction services by specifically examining how dimensions of anger—particularly trait anger and anger suppression—interact with burnout in this professional group. While previous studies have documented high levels of burnout among healthcare workers, this research adds a novel focus on the emotional mechanisms underpinning that burnout, identifying anger as not merely a symptom but a dynamic contributor. Through a rigorous psychometric assessment using the STAXI-2 and MBI in a defined and underexplored geographic context (Calabria and Sicily), this study sheds light on emotional dynamics rarely analyzed together in frontline addiction professionals.

### 4.1. Burnout Among SERD Operators

The data from this study confirm that SERD operators are at a heightened risk for burnout, particularly in the dimension of emotional exhaustion. The mean emotional exhaustion score of 32.7 is consistent with burnout levels reported in other high-stress healthcare settings, such as emergency departments, oncology units, and mental health services [47,48]. Emotional exhaustion, which reflects feelings of being emotionally depleted and overwhelmed by the demands of the job, was especially pronounced among female participants. This gender difference is not unique to addiction services and has been well-documented in the burnout literature. Research suggests that women in healthcare may experience greater emotional exhaustion due to gendered expectations around caregiving, higher levels of empathy, and the emotional labor associated with their roles [26,49]. 

The relatively high levels of depersonalization reported by participants, particularly among those with more than ten years of service, highlight the potential for long-term exposure to emotionally taxing patient interactions to result in detachment or cynicism. This is consistent with Maslach’s burnout theory, which posits that depersonalization is a defense mechanism that individuals develop in response to chronic emotional exhaustion, allowing them to cope with the emotional demands of their work by distancing themselves from their clients [29,50]. However, while this detachment may temporarily protect workers from emotional distress, it can also undermine the therapeutic relationship and the quality of care provided to patients [51]. 

Interestingly, despite the high levels of emotional exhaustion and depersonalization, participants in this study reported relatively high levels of personal accomplishment. This finding may reflect the intrinsic rewards associated with helping individuals overcome addiction, a deeply challenging but potentially highly satisfying field of work. Workers in addiction services often report that witnessing patient recovery, even if rare, provides a strong sense of personal efficacy and professional fulfillment. However, it is also possible that the high personal accomplishment scores in this study may reflect a form of cognitive dissonance reduction, where workers compensate for feelings of exhaustion and detachment by emphasizing their professional achievements [52]. 

### 4.2. The Role of Anger in Burnout

One of the most significant contributions of this study is the demonstration of the role that anger, particularly trait anger and anger suppression (anger-in), plays in exacerbating burnout. The positive correlation between trait anger and emotional exhaustion aligns with existing research that identifies anger as a maladaptive emotional response that can intensify stress and fatigue [53]. Trait anger reflects a stable disposition to experience anger across a variety of situations, and in high-stress environments such as addiction services, this predisposition may lead to chronic emotional arousal, which in turn depletes emotional resources and contributes to exhaustion [54]. 

Moreover, the finding that anger-in was a significant predictor of depersonalization provides new insights into the emotional dynamics of burnout in addiction services. Anger suppression has been linked to a range of negative psychological outcomes, including depression, anxiety, and burnout [55,56]. When anger is suppressed rather than expressed or processed, it may accumulate over time, leading to emotional disengagement from patients as a means of coping with unresolved frustration and irritation [57]. In the context of addiction services, where patients may exhibit challenging behaviors such as relapse or resistance to treatment, the chronic suppression of anger could lead to feelings of helplessness and cynicism, further fueling depersonalization [58]. 

The role of anger control, on the other hand, emerged as a protective factor in this study. Participants who reported higher levels of anger control were less likely to experience burnout, particularly in terms of personal accomplishment. This finding supports the broader literature on emotional regulation, which posits that individuals who are able to manage their emotional responses to stress are better equipped to maintain psychological well-being and professional efficacy [59]. Anger control, in this context, may involve recognizing and addressing sources of frustration in a constructive manner, thereby preventing the accumulation of negative emotions that can contribute to burnout [60]. 

### 4.3. Underlying Mechanisms: Emotional Labor and Coping Strategies

To understand the mechanisms through which anger contributes to burnout, it is important to consider the concept of emotional labor, which refers to the process of managing one’s emotions to meet the demands of a job [61]. Healthcare professionals, particularly those working in addiction services, are often required to display empathy, patience, and understanding, even in the face of challenging or emotionally charged interactions. This emotional regulation can be particularly taxing when workers are simultaneously experiencing high levels of anger, which they may feel compelled to suppress in order to maintain a professional demeanor [62].

The emotional labor involved in addiction treatment is further complicated by the chronic nature of substance use disorders and the frequent relapses that characterize recovery [8]. When patients relapse, healthcare workers may experience feelings of frustration or helplessness, which, if not adequately addressed, can contribute to the development of burnout [10]. The suppression of anger in these situations, as indicated by the high anger-in scores in this study, may reflect a coping strategy aimed at maintaining professional composure, but over time, it can lead to emotional exhaustion and depersonalization [63]. 

Previous research has identified several coping strategies that may mitigate the impact of emotional labor and anger on burnout. For example, problem-focused coping, which involves taking action to address the sources of stress, has been shown to reduce burnout by enabling individuals to feel more in control of their work environment [64]. In contrast, emotion-focused coping, such as the suppression of anger, is associated with higher levels of burnout, as it does not address the underlying sources of emotional distress [65]. These findings suggest that interventions aimed at improving emotional regulation and promoting adaptive coping strategies may be effective in reducing burnout among SERD operators.

### 4.4. Implications for Interventions and Workplace Policies

The results of this study have several important implications for the development of interventions and policies aimed at reducing burnout and promoting emotional well-being among addiction service workers. Given the strong relationship between anger and burnout, particularly in the dimensions of emotional exhaustion and depersonalization, interventions that focus on emotional regulation and anger management are likely to be beneficial. Programs such as mindfulness-based stress reduction (MBSR), which have been shown to improve emotional regulation and reduce burnout in healthcare workers, could be particularly effective in helping SERD operators manage the emotional demands of their work [66,67]. 

In addition, workplace policies that promote organizational support and reduce the systemic stressors that contribute to burnout should be prioritized. For example, reducing caseloads, providing opportunities for professional development, and fostering a supportive organizational culture have all been identified as key factors in preventing burnout [68]. Providing access to mental health services for staff, including counseling and stress management programs, could also help to alleviate the emotional burden of working in addiction services [21]. 

Another important consideration is the role of supervision and peer support in mitigating the effects of burnout and anger. Research has shown that healthcare workers who receive regular supervision and have access to peer support are less likely to experience burnout, as these resources provide opportunities for emotional processing and professional reflection [17]. Creating spaces where SERD operators can discuss their emotional experiences, including feelings of frustration and anger, without fear of judgment or professional repercussions, may help to reduce the tendency to suppress negative emotions and promote healthier emotional regulation [5]. 

Finally, preventive measures should be implemented at the organizational level to reduce the risk of burnout before it becomes entrenched. This may involve regular assessments of staff well-being, early identification of burnout symptoms, and the development of tailored interventions for at-risk employees [69]. By taking a proactive approach to burnout prevention, organizations can not only improve the mental health of their staff but also enhance the quality of care provided to patients.

## 5. Conclusions

The present study offers a focused contribution to the literature on burnout and emotional dysregulation among addiction service professionals, a population historically underrepresented in empirical research. By exploring the specific dimensions of anger (trait, suppression, control) and their predictive value for burnout outcomes in a regional sample of SERD workers in Calabria and Sicily, our results provide a targeted framework for understanding how emotional regulation strategies relate to mental health risks in high-stress care settings. The findings suggest actionable paths for individual and systemic interventions.

### 5.1. Key Findings and Their Implications

This study confirmed the high prevalence of emotional exhaustion among SERD operators, echoing patterns observed in other high-stress medical contexts [28,44]. It also revealed a strong correlation between trait anger and emotional exhaustion, and highlighted anger suppression (anger-in) as a key factor in depersonalization—providing an empirical basis for treating anger not only as a symptom, but as a mechanism contributing to burnout [16,20,41,58]. 

The study also shows how emotional labor, particularly in roles demanding sustained empathy, contributes to burnout when anger is unmanaged [61,62]. This is compounded by role-specific pressures in addiction services, where relapses and patient resistance are frequent [13,48]. Despite high stress, participants reported a strong sense of personal accomplishment—possibly indicating the compensatory value of intrinsic motivation or cognitive reframing [52]. 

Furthermore, our data support the notion that anger control is a protective factor. Individuals who demonstrate better anger regulation also report greater professional fulfillment [59,60]. These dynamics have been underexplored in the context of addiction services. Variation in burnout experiences based on professional role was consistent with findings from other sectors (e.g., education, law enforcement, forestry), suggesting that interventions should be adapted based on specific job demands [70,71,72]. 

### 5.2. Practical Implications for Interventions

The results support the implementation of individual-focused programs such as mindfulness-based stress reduction (MBSR) and cognitive behavioral therapy (CBT), which have been shown to be effective in helping healthcare workers regulate emotions and reduce burnout [72,73,74]. 

Anger management training should be integrated into professional development programs to help SERD workers identify and respond to emotional triggers constructively [75]. At the organizational level, efforts should focus on reducing workload, improving access to mental health resources, and promoting peer support to alleviate systemic sources of burnout [17,21,68]. 

### 5.3. Policy Implications

This study reinforces the need for national and regional policies that formally recognize emotional regulation training as a core competency in addiction services. Support for evidence-based interventions and the institutionalization of burnout screening could promote more sustainable treatment environments [76,77,78]. 

### 5.4. Limitations of the Study

As a cross-sectional study, causality cannot be inferred [71,72,79]. The sample is regionally bound (Calabria and Sicily), and although representative of those territories, generalizability should be cautiously considered [80]. Other emotional states (e.g., anxiety, guilt) were not examined but could meaningfully contribute to burnout dynamics [81]. 

### 5.5. Ethics Statement

This study did not require formal approval by an institutional ethics committee, as it involved the administration of standardized, non-invasive psychodiagnostic instruments (Maslach Burnout Inventory and STAXI-2) routinely used in occupational and clinical research. According to Italian guidelines, such observational designs are exempt from mandatory ethics board submission. The research complied fully with the Declaration of Helsinki, and each participant gave written informed consent.

### 5.6. Future Research Directions

Future research should adopt longitudinal methods to clarify temporal relationships between emotional dysregulation and burnout, and assess the efficacy of targeted interventions such as anger management programs and MBSR.

Additionally, organizational factors (e.g., supervision style, institutional support) should be analyzed as moderators or mediators in the burnout-anger relationship [82]. Finally, it is be critical to assess how burnout and anger affect patient outcomes, with implications for public health, workforce sustainability, and care quality.

## Data Availability

The data supporting the findings of this study are available upon reasonable request from the corresponding author. Due to ethical restrictions, individual-level data cannot be publicly shared.
